# Editorial: Suicide and Related Behaviour

**DOI:** 10.3389/fpsyt.2021.676487

**Published:** 2021-04-13

**Authors:** Gonzalo Martínez-Alés, María Luisa Barrigón, Jorge López-Castroman, Enrique Baca-García

**Affiliations:** ^1^Department of Epidemiology, Columbia University Mailman School of Public Health, New York, NY, United States; ^2^Department of Psychiatry, La Paz University Hospital, Madrid, Spain; ^3^Department of Psychiatry, Fundación Jiménez Díaz University Hospital, Madrid, Spain; ^4^Department of Psychiatry, Universidad Autónoma de Madrid School of Medicine, Madrid, Spain; ^5^Nimes University Hospital, Nimes, France; ^6^Institute of Functional Genomics, University of Montpellier, CNRS, INSERM, Montpellier, France

**Keywords:** suicide, attempted suicide, mediation, global mental health, data science

Suicide claims almost 1 million lives globally every year. Understanding and preventing suicidal behaviors and death by suicide is a largely unmet need: despite substantial efforts, suicide remains the second leading cause of death among youth and, over the last two decades, suicide mortality rates have increased in several regions across the globe (1). For instance, in the United States, suicide is the only leading cause of death that has not diminished over the last two decades—alongside opioid overdose, an entity that is closely related to suicide (2). The impact of suicide is far-reaching and affects families and communities over generations. Advancing suicidology is an urgent public health challenge.

Understanding suicidal behaviors is complex, as they emerge in the context of a complex multilevel chain of causation that includes poorly clarified neurobiological pathways and dynamic individual- and group-level risk and protective factors, interacting in a nuanced balance. Accordingly, despite substantial efforts, advancement of suicide risk stratification, prevention, and treatment tools over the last decades has been limited. Key limitations driving this phenomenon have been pointed out elsewhere and include, among others, (i) the fact that most research on suicide comes from a relatively small number of high-income countries, even though most people who die by suicide live in low and middle-income countries (3), (ii) traditional ethical reasons to systematically exclude suicidal individuals from clinical studies (4), or (iii) technical limitations, such as a generalized lack of enough detailed information in large prospective cohorts or a poor uptake of modern causal mediation epidemiological methods.

In this special topic on Suicide and Related Behavior, we sought to overcome traditional limitations of suicide research by expanding our scope to innovative approaches, both in terms of methodological tools and study populations. Here we provide a brief overview of the contents and highlights of the special topic.

A set of six studies take a mediation/moderation causal analysis approach to suicidal behaviors. Dal Santo et al. examine the role of impulsivity as a mediator in the association between early traumatic experiences and suicidal behaviors in a sample of 190 depressed patients. Using a simple mediation approach, they find results suggesting that impulsivity may play a mediating role in the association, an important finding with substantial potential implications for individual-level prevention. Rubio et al. adopt Hayes's approach to mediation, based on structural equation modeling, to prove the hypothesis that negative affect and suicide attempts are mediated by suicidal ideation in a large representative sample of Chilean high school students. A large study including almost 500 Chinese students assessed 4 years after the Ya'an earthquake by Liu et al. also uses a structural equation modeling approach to study the association between self-compassion and suicide risk, finding that positive and negative self-compassion, respectively, reduce and increase suicide risk, and that gratitude and post-traumatic stress disorder may be salient mediators. Mészáros et al. examine a sample of 363 (202 suicidal and 163 non-suicidal) adolescents to examine another highly relevant and novel variable, namely pathological internet use, and test whether it is associated with non-suicidal self-injury directly or through a series of psychopathological domains, finding no apparent link between self-injury and pathological internet use. Examining a large sample of Chilean adolescents, Núñez et al. investigate the associations between depressive symptoms, psychotic experiences, and suicidal ideation, describing patterns of association between specific symptoms from each of the three domains. Last, also using a randomly selected sample of adolescents from Santiago de Chile, another study by Rubio, Oyanedel, Cancino et al. examines social support and substance (alcohol, marijuana, and other illicit drugs) use as moderators of the relationship between depressive symptoms and suicidal ideation. They report that social support, regardless of whether provided by peers, family, or school; and alcohol use both moderate the depression-ideation association.

Two studies build on the rich tradition of observational research to identify salient correlated of suicidal risk. Lin et al. also focus on impulsivity. Their ingenious experiment couples a two-choice oddball paradigm with emotional stimuli and electroencephalogram recording to characterize participants' psychophysiological profile. Suicidal ideation is found to be associated with certain specific changes in behavioral inhibitory control in response to emotional information. These results suggest that what the authors call an “emotion-impulsivity framework” in information processing may be associated with suicidal ideation. Harnod et al., using Taiwan's nationwide population-based databases, examine more than 600,000 adults with sleep-disordered breathing to examine the prospective association between head trauma and suicide risk. Their finding of a 2-fold increase in risk among individuals with a history of head trauma, after adjusting for a comprehensive set of potential confounders, is accompanied by detailed examination of other risk factors, including a potential synergistic effect with age, greatly enhancing understandability of potential implications.

Over the last years, evidence supporting brief interventions, including contact and psychotherapeutic interventions, has somewhat brought about a renovated hope in suicide prevention intervention research. Here, in a pilot study featuring 26 adolescents, Haruvi Catalan et al. examine the foundations of an ultra-brief crisis intervention based on Interpersonal Therapy for high-risk children and adolescents. Their preliminary results are promising and suggest treatment safety, feasibility, and acceptability. Also, the emerging role of people with lived experiences in discussions around mental health is changing the current paradigm of knowledge production in psychiatric research. Schilling et al. report qualitative assessments of a group of seven adolescents serving as research guides in the development of Project Clan, a technology-based intervention to reduce adolescent suicide in Chile. They provide critical insights to understand the specifics underlying adolescent suicide, emphasizing the importance of generating anonymous, secure spaces for self-expression online. Their experience adds to existing evidence supporting incorporation of people with lived experiences in every step of the development of mental health interventions. Last, a meta-research study by Bittar et al. reviews the wide range of studies related to suicide conducted using the Clinical Record Interactive Search, a large data repository from London. They provide a critical appraisal of studies using only structured information and highlight the most important milestones in the use of non-structured clinical records for epidemiological research using natural language processing and data mining techniques. This study should prompt discussion, as it covers in detail a hotly debated topic in research using electronic health records—namely use of automatized data extraction and analysis methods.

Suicide is preventable tragedy. We thank the research teams featured in this special topic for their work and encourage them to continue generating knowledge to critically enhance our understanding of suicide, its causes, and its prevention. We hope that readers of the Journal will find this special topic timely and interesting, and that the results included here help guide decision-making at several levels.

## Author Contributions

All authors conceived and developed the presented idea, and contributed to the writing of the final manuscript equally.

## Conflict of Interest

The authors declare that the research was conducted in the absence of any commercial or financial relationships that could be construed as a potential conflict of interest.
